# Ab Initio Study of Structural and Electronic Properties of (ZnO)_*n*_ “Magical” Nanoclusters *n* = (34, 60)

**DOI:** 10.1186/s11671-017-1848-8

**Published:** 2017-01-25

**Authors:** Rostyslav Bovhyra, Dmytro Popovych, Oleg Bovgyra, Andrew Serednytski

**Affiliations:** 1 Pidstryhach Institute for Applied Problems of Mechanics and Mathematics NAS Ukraine, Naukova 3-B, Lviv, Ukraine; 2 Faculty of Physics, Ivan Franko National University of Lviv, Kyrylo and Mefodiy 8, Lviv, 79005 Ukraine

**Keywords:** Structure, Electronic properties, ZnO nanoclusters, The density functional theory

## Abstract

Density functional theory studies of the structural and electronic properties of nanoclusters (ZnO)_*n*_ (*n* = 34, 60) in different geometric configurations were conducted. For each cluster, an optimization (relaxation) of structure geometry was performed, and the basic properties of the band structure were investigated. It was established that for the (ZnO)_34_ nanoclusters, the most stable are fullerene-like hollow structures that satisfy the rule of six isolated quadrangles. For the (ZnO)_60_ nanoclusters, different types of isomers, including hollow structures and sodalite-like structures composed from (ZnO)_12_ nanoclusters, were investigated. It was determined that the most energetically favorable structure was sodalite-type structure composed of seven (ZnO)_12_ clusters with common quadrangle edges.

## Background

Wide-gap semiconductors are perspective materials to use in optoelectronic systems, ultraviolet lasers, field emitters, and other devices of new generation. It is said that not only the composition but also the nature of the nanostructures give new properties to the material. Atomic clusters and fullerenes are the building blocks of the new nanostructured materials which are a subject of intensive research with the prospect of applications in optoelectronics. Special interest is given to the clusters of zinc oxide which, with its variety of interesting physical and chemical properties, such as anisotropic crystalline structure, semiconducting properties even with a wide band gap, amphoteric chemical properties, piezoelectric properties, biocompatibility, and high exciton energy, is quite unique [1, 2]. A large number of studies have been devoted to understand its structure, processes of formation and properties, and the behavior of its nanoparticles [3–5]. Thin films and nanostructures based on ZnO, are candidates for creating ultrathin displays, UV emitters and switches [6, 7], and gas sensors [8].

The main methods of studying the electronic properties of atomic clusters are quantum mechanics methods, such as restricted and unrestricted Hartree-Fock method, the density functional theory, and molecular dynamics. To address this problem is to use theoretical methods to study model clusters, particularly in structures that lie between molecular and bulk. Nonetheless, the structure design still allows for many geometric possibilities to exist, and it is challenging to find a true global minimum energy structure.

Numerous theoretical studies of (ZnO)_*n*_ clusters have explored optimized geometries for a range of cluster sizes, and a prevalent theoretical observation shows that a fullerene-like structures are more stable in the case for smaller-sized clusters, while a wurtzite-like structure shows increased stability for larger clusters [9]. A core-cage structure for (ZnO)_34_ has been proposed as the most stable in [10, 11], while [12] have predicted the hollow cage structures formed by (ZnO)_2_ squares and (ZnO)_3_ hexagons. In the case of (ZnO)_60_, the studies [13, 14] revealed an energetically preferred sodalite motif, while nested cage configuration was predicted to be the most stable in [10, 11]. Such differences indicate that there is a strong dependence of the calculated binding energy on the details of the computational framework adopted.

This paper presents a theoretical investigation of structural and electronic properties of clusters (ZnO)_*n*_ (*n* = 34, 60), within the density functional theory, in different geometric configurations to establish which type of structure is the most energetically favorable.

## Methods

Ab initio calculations within density functional were performed, which have been successfully used for studying properties of nanoscale structures such as nanotubes and nanowires [15–18]. For structural models, the optimization (relaxation) of the geometry (finding the equilibrium of ions coordinates, in which the full electronic energy of the system is minimal) was carried. Optimization was calculated using the effective algorithm of delocalized internal coordinates [19]. The convergence of the relaxation procedures deemed reached when the magnitude of forces acting on atoms was less than 0.05 eV/Å.

For describing the exchange-correlation energy of the electronic subsystem, the generalized gradient approximation (GGA) in a parameterization of Perdew, Burke, and Ernzerhof was used [20]. It is known that the use of this approach in the calculation leads to underestimation of the quantitative value of the binding energy. On the other hand, an alternative description of the exchange-correlation interaction within local density approximation (LDA) leads to overestimation of the energy values compared to the experimental data. Using GGA in this paper makes it possible to argue that if calculation results say that the cluster model is stable, then the real system will be stable as well. Electronic functions of electrons were divided in the basis of atomic orbitals, including *d*-orbitals. Core electrons had been described using effective potential with regard to relativistic corrections. Integration in the first Brillouin zone was conducted in the Monkhorst-Pack k-point set [21].

## Results and Discussion

**Fig. 1:**
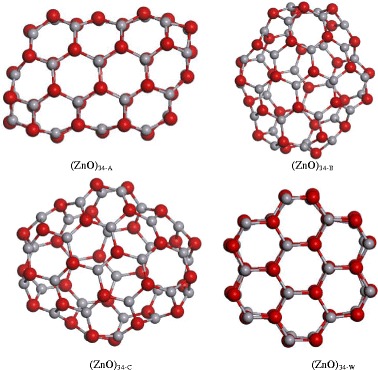
Optimized structures of (ZnO)_34_ nanoclusters performed at the DFT/GGA level of theory

**Fig. 2:**
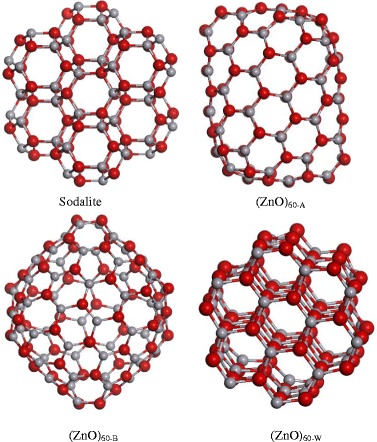
Optimized structures of (ZnO)_60_ nanoclusters performed at the DFT/GGA level of theory

In order to determine the most stable structure for “magic” clusters (ZnO)_34_ (Fig. 1) and (ZnO)_60_ (Fig. 2), we examined a number of isomers. Among them were hollow fullerene-like structures and cage structures which met the rule of six isolated quadrangles.

There were also sodalite-like structures composed of structural units of (ZnO)_12_. For each cluster, geometry optimization was performed and band structure properties were analyzed.

The binding energy of ZnO cluster as per formula unit was calculated using the formula [22]:

$$ {E}_{\mathrm{b}} = E\ \left(\mathrm{Zn}\right) + E\left(\mathrm{O}\right)\ \hbox{--}\ 1/ n*{\mathrm{E}}_n, $$

where *n* is the number of ZnO molecules in a cluster, *E* (Zn) and *E* (O) the basic energy states of atoms of Zn and O, and *E*_*n*_ the total energy of a (ZnO)_*n*_ cluster.

In Table 1, the geometry parameters of (ZnO)_34_ and (ZnO)_60_ nanoclusters are presented. They include minimal and maximal interatomic distances (*d*, Å) between Zn and O atoms in quadrangles and hexagons, respectively, diameter (distance between the edges of a cluster *D*, Å) of the clusters, and range of values for angles in quadrangles and hexagons.

**Table 1:** Geometry parameters of (ZnO)_34_ and (ZnO)_60_ nanoclusters

Isomer	d, Å in quadrangles	d, Å in hexagons	D, Å	α, in quadrangles	α, in hexagons
(ZnO)_34-28_	1.945–1.984	1.886–1.984	14.827	84.761–91.960	113.612–132.058
(ZnO)_34-43_	1.938–1.994	1.881–1.994	13.013	85.315–93.708	117.331–122.628
(ZnO)_34-15_	1.938–1.992	1.912–1.992	13.018	84.791–93.952	107.145–128.283
(ZnO)_60-sodalite_	1.931–2.284	1.884–2.284	15.659	83.927–97.183	106.583–134.193
(ZnO)_60-25_	1.963–1.968	1.896–1.968	16.342	85.782–92.117	109.977–129.234
(ZnO)_60-24_	1.964–1.970	1.890–1.970	16.112	85.622–92.846	110.167–129.060

For all clusters, the maximum value of interatomic distance between Zn and O atoms is set for joint edge between quadrangle and hexagon. For angle values, we established that smaller angles correspond to oxygen atoms and bigger angles correspond to zinc atoms.

In Table 2, we present the properties of electronic spectra of (ZnO)_34_ and (ZnO)_60_ nanoclusters.

**Table 2:** Electronic properties of (ZnO)_34_ and (ZnO)_60_ nanoclusters

Isomer	*E*_total_/ZnO, eV	Δ*E*/ZnO, eV	*E*_b_/ZnO, eV	*E*_g_, eV
(ZnO)_34-A_	–50461.66	0	–6.764	2.275
(ZnO)_34-B_	–50461.64	0.02	–6.748	2.151
(ZnO)_34-C_	–50461.62	0.04	–6.724	2.048
(ZnO)_34-W_	–50461.54	0.12	–6.645	1.124
(ZnO)_60-sodalite_	–50461.744	0	–6.847	1.93
(ZnO)_60-A_	–50461.734	0.01	–6.836	2.184
(ZnO)_60-B_	–50461.732	0.012	–6.835	2.4
(ZnO)_60-W_	–50461.699	0.045	–6.802	0.982

**Fig. 3:**
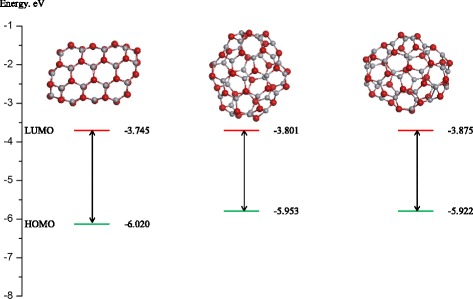
HOMO and LUMO levels calculated using GGA functional for nanoclusters (ZnO)_34_

In the first column, we have total energy per formula unit of each isomer, second column is the difference between total energies with respect to the isomer with lowest energy separately for (ZnO)_34_ and (ZnO)_60_, third column is binding energy per formula unit, and band gap energy is given in the fourth column. Analysis of the energy values shows that the most energetically favorable (ZnO)_34_ nanoclusters are fullerene-like hollow structures. All such structures that meet the rule of isolated quadrangles are close in value of binding energy. The calculated values are larger than the bulk-binding energy of ZnO (−7.52 eV per formula unit) as expected due to surface energy effects. Confirmation of high stability for these clusters is the higher values of band gap between the highest occupied molecular orbital (HOMO) and lowest unoccupied molecular orbital (LUMO) because such clusters are chemically inert (Fig. 3).

**Fig. 4:**
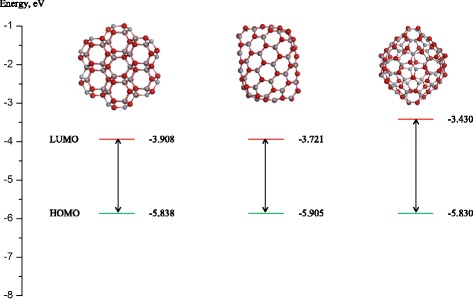
HOMO and LUMO levels calculated using GGA functional for nanoclusters (ZnO)_60_

In the case of (ZnO)_60_ nanoclusters, we confirmed that the most stable among them is the sodalite structure which is built from 7 (ZnO)_12_ nanoclusters with joint quadrangle edges. In previous studies [23], it was shown that the (ZnO)_12_ cage-like structure (truncated octahedron) proved to be very stable compared to other small (ZnO)_*n*_, suggesting that it can be used as a building block for creating ZnO nanostructures. The values for HOMO and LUMO for sodalite, as well as the other (ZnO)_60_ structural isomers, are presented in Fig. 4.

**Fig. 5:**
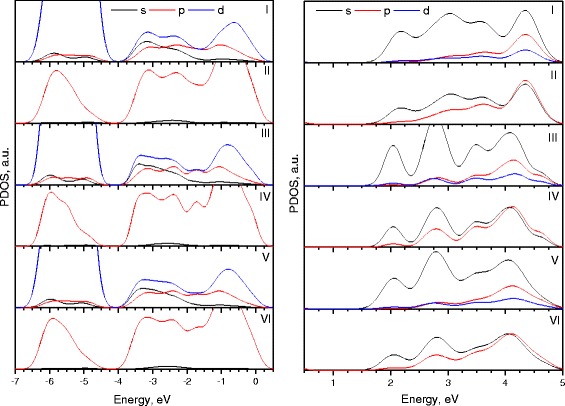
Partial densities of states of (ZnO)_34-A_ (I, II), (ZnO)_34-B_ (III, IV), and (ZnO)_34-C_ (V, VI) nanoclusters

In Fig. 5, partial densities of states from the contributions of different orbital components for each (ZnO)_34_ nanocluster for valence band (left) and conduction band (right) are presented. Graphs I, III, and V demonstrate s, p, and d states of Zn atoms; graphs II, IV, and VI correspond to s and p states of O atoms.

The valence band of each cluster between −7.0 and −4.0 eV consist mainly from 3d states of Zn and O 2p states. The bands between −4.0 and 0 eV are composed from O 2p states, Zn 3d states, and in smaller scale, Zn 3p and 3s states. The conduction band, on the other hand, between 1 and 5 eV consists mainly from Zn 4s and O 2p and O 2s states.

**Fig. 6:**
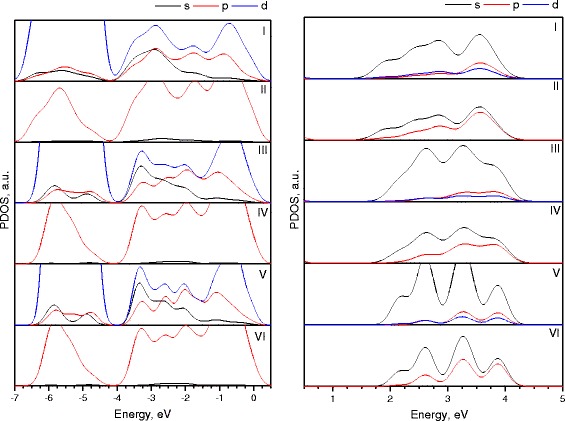
Partial densities of states of (ZnO) sodalite (I, II), (ZnO)_60-A_, (III, IV), and (ZnO)_60-B_ (V, VI) nanoclusters

Figure 6 shows partial densities of states from the contributions of different orbital components for each (ZnO)_60_ nanocluster for valence band (left) and conduction band (right). Graphs I, III, and V show s, p, and d states of Zn atoms, and graphs II, IV, and VI correspond to s and p states of O atoms.

The valence band of each cluster between −7.0 and −4.0 eV, like in the case with (ZnO)_34_ nanoclusters, is composed from 3d states of Zn and O 2p states. The bands between −4.0 and 0 eV consist mainly from O 2p states, Zn 3d states, and in smaller scale, Zn 3p and 3s states. The conduction band between 1 and 5 eV consists mainly from Zn 4s and O 2p and O 2s states.

## Conclusions

Density functional theory studies of the structural and electronic properties of (ZnO)_*n*_ (*n* = 34, 60) nanoclusters were performed. Optimization of structure geometry, as well as the band structure research, was performed. It was established that for the (ZnO)_34_ nanoclusters, the most stable are the fullerene-like hollow structures that satisfy the rule of six isolated quadrangles. For the (ZnO)_60_ nanoclusters, different types of isomers, including hollow structures and sodalite-like structures composed from (ZnO)_12_ nanoclusters, were investigated. It was determined that the most energetically favorable structure was the sodalite-type structure composed of seven (ZnO)_12_ clusters with common quadrangle edges.
